# Postnatal structural development of mammalian Basilar Membrane provides anatomical basis for the maturation of tonotopic maps and frequency tuning

**DOI:** 10.1038/s41598-021-87150-w

**Published:** 2021-04-07

**Authors:** Tomomi Tani, Maki Koike-Tani, Mai Thi Tran, Michael Shribak, Snezana Levic

**Affiliations:** 1 Marine Biological Laboratory, Eugene Bell Center, Woods Hole, MA USA; 2 Biomedical Research Institute, National Institute of Advanced Industrial Science and Technology, Ikeda, Osaka Japan; 3 Integrated Frontier Research for Medical Science Division, Institute for Open and Transdisciplinary Research Initiatives (OTRI), Osaka University, Suita, Osaka Japan; 4 College of Engineering and Computer Science, VinUniversity, Gia Lam District, Hanoi, Vietnam; 5 Sensory Neuroscience Research Group, School of Pharmacy and Biomolecular Sciences, University of Brighton, Huxley Building, Brighton, BN2 4GJ UK; 6 Brighton and Sussex Medical School, University of Sussex, Brighton, BN1 9PX UK

**Keywords:** Auditory system, Development of the nervous system, Diseases of the nervous system, Sensory processing, Developmental biology, Neuroscience, Imaging

## Abstract

The basilar membrane (BM) of the mammalian cochlea constitutes a spiraling acellular ribbon that is intimately attached to the organ of Corti. Its graded stiffness, increasing from apex to the base of the cochlea provides the mechanical basis for sound frequency analysis. Despite its central role in auditory signal transduction, virtually nothing is known about the BM’s structural development. Using polarized light microscopy, the present study characterized the architectural transformations of freshly dissected BM at time points during postnatal development and maturation. The results indicate that the BM structural elements increase progressively in size, becoming radially aligned and more tightly packed with maturation and reach the adult structural signature by postnatal day 20 (P20). The findings provide insight into structural details and developmental changes of the mammalian BM, suggesting that BM is a dynamic structure that changes throughout the life of an animal.

## Introduction

The basilar membrane (BM), of the mammalian cochlea serves as the anatomical substrate for frequency selectivity, fundamental function of hearing, whereby incoming sounds are decomposed into specific frequencies. The width of the BM decreases, while its thickness and stiffness increase from the apex to base of the cochlea^1^. Together these spatially varying characteristics of the BM confer frequency-to-place (tonotopic) organization to the cochlea, with the base tuned to high frequency and the apex to low-frequency sounds. This passive, mechanical, tonotopic frequency map provides a basis for neural encoding of frequency information to higher auditory processing centers^1,2^, a process enhanced by the non-linear cochlear amplifier^3^. The ability for sound to be encoded correctly with fine frequency resolution is essential for auditory perception, which guides appropriate behavioral responses, including the ability to understand speech^4,5^.

The BM is a specialized layer of extracellular matrix (ECM) sandwiched between the overlying sensory epithelium, containing the sensory hair cells and supporting cells, and an underlying layer of tympanic border cells^6^. BM structural components are most probably secreted from the underlying mesothelial cells^7,8^. Early anatomical studies using electron microscopy revealed that the mature BM is composed of radial collagenous filaments, 10–12 nm in diameter, embedded in a matrix of ground substance^9–11^. The BM filaments are composed primarily of laminin-β2 and collagen types II and IV^12–16^. The collagenous fibers have been associated with several glycoproteins, including Fibronectin and Tenascin^16^. More recently, Emilin2 was identified in the BM^17^ and was shown to affect collagen fibril arrangement, and thereby to affect its elastic properties and frequency tuning bandwidth^18^. This finding agrees with the generally accepted mechanical basis of frequency tuning in the cochlea that depends on the apical basal stiffness gradient of the BM which, in turn, depends largely on its structural organization^1,3^.

A remarkable feature of the basal, high frequency, cochlea region of altricial mammals, which occurs in the third week of development, is the upward shift in the frequency of the tone most effective in eliciting gross cochlear potentials^19–21^. Moreover, auditory nerve responses become more sensitive and sharply tuned^22,23^. Neural frequency tuning originates as a consequence of BM vibrations^1,3^ and the developmental upward frequency shift in the tonotopic neural frequency map of the cochlea has been demonstrated to be associated with an upward shift in the BM vibration frequency map^24–26^. Concomitantly, the cochlear partition in the basal turn of the cochlea becomes stiffer^27^. Developmental change in stiffness of the cochlear partition could involve changes in several structures. For example the dimensions of the organ of Corti and thickness of the radial fibre bands of the BM increase during development consistent with the upward shift in the BM frequency, such as increases in the thickness of the radial fibre bands within the BM frequency map^22,28^. There is also a developmental increase in the density of proteoglycans^29^, increased proliferation of tension fibroblasts in the spiral ligament^22,30^ and maturation of the pillar cells of the organ of Corti^31^.

Currently, it is unknown if these developmental changes in the structure of the cochlear partition occur independently of each other or are interdependent. As a first step to understanding the basis for developmental changes in the stiffness of the cochlear partition and their possible interdependence, we exploited polarized light microscopy techniques using acutely isolated BM in wild type (emilin2^+/+^) and *Emilin-2*^*-/-*^ (emilin2^-/-^) mice. Polarized light microscopy provides a label free, non-invasive, sensitive tool to analyze the alignment of fine structural signatures in cells or tissues^32^. All tissues, cells, and organelles that include extensive membranous structures—such as cochlear ECMs can exhibit birefringence (refractive index anisotropy) that are characteristic of their molecular architecture (such as collagen fibrils, stress fibers made of filamentous actin and myosin, and microtubules (Supplementary Fig. 1). New polarized light microscopy techniques (LC-PolScope and Polychromatic PolScope^33–35^) can represent the optical axis of birefringence and map the measured orientation. Thus, quantitative information available in PolScope images allowed for unique insights into the architectural dynamics of the BM.

This is the first report to show that the BM undergoes dynamical changes during development and maturation. BM filaments become thicker, more tightly packed and radially ordered with maturation, while emilin2 mediated signaling is necessary for the structural maturation of the developing mammalian BM with consequences for the structure and the mechanical responses of the cochlear partition^18^.

## Results

### Using polarized light microscopy to study basilar membrane structure

**Figure 1:**
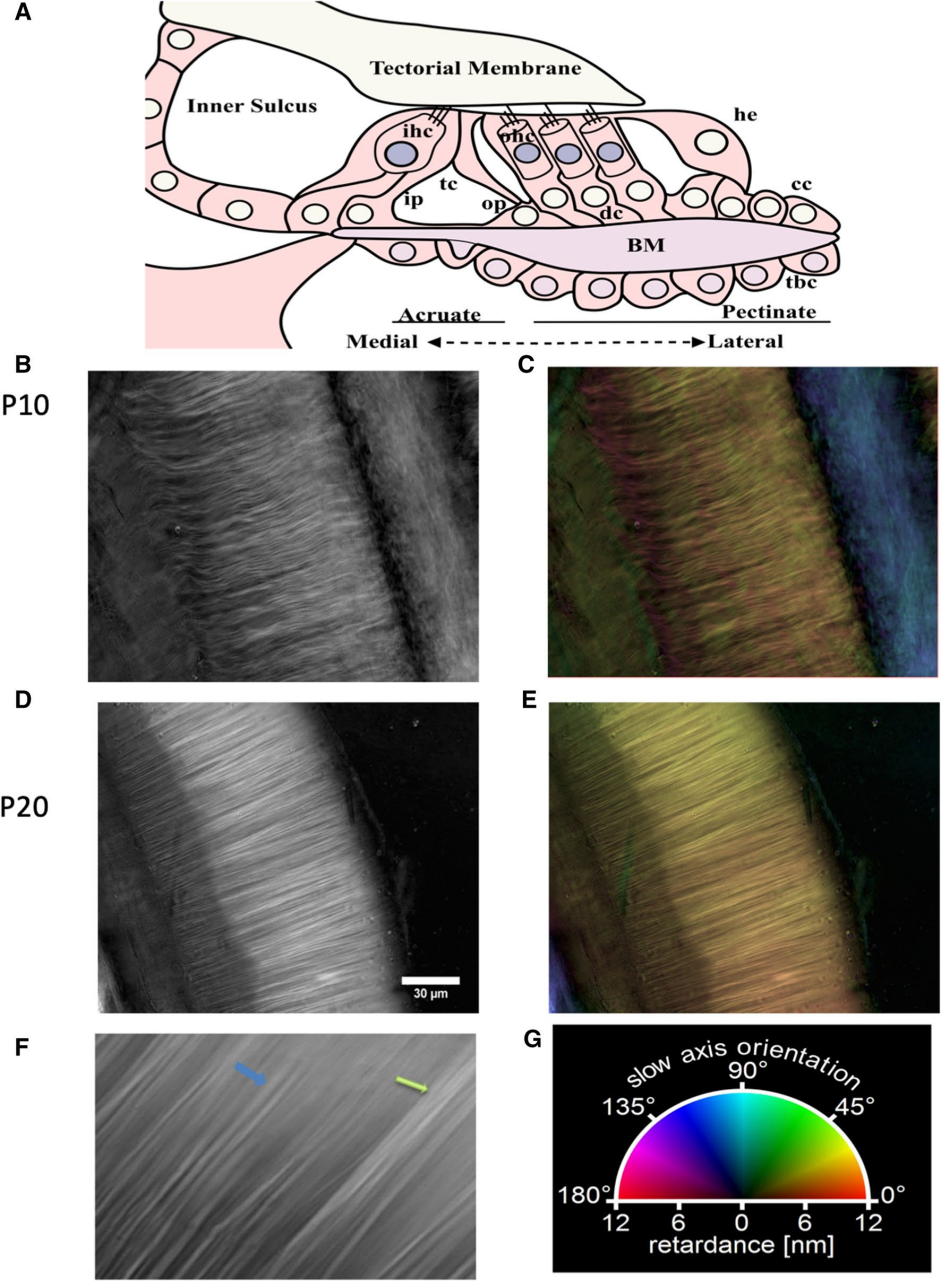
Polarized light images reveal differences in structural signature during basilar membrane development and maturation. (**A**) Schematic of the Organ of Corti (**B**,**D**) Retardance images of the midturn BM at P10 (**B**) and P20 (**D**). A retardance image represents the measured magnitude of refractive index anisotropy and is the first of the two calculated images in a LC-PolScope image stack. **(C**, **E**) Pseudo color composition of retardance and orientation of the samples in (**B**) and (**C**). The orientation image is the second image in an LC-PolScope stack and is used to determine the hue, while the retardance image is used to determine the brightness of the pseudo-color image. The overall hues of (**C**) and (**E**) represents the slow axis of orientation (see color wheel (**G**). The color variation in (**C**) and (**E**) reveals a great degree of refractive index anisotropy. (**F**) Enlarged section showing the individual fibers, the smallest detectable single unit (blue arrow) and fiber bundles (green arrow). Scale bar length same as shown in (**D**): 5 μm. (**G**) Color wheel shows a visual vectorial representation of orientations. A color wheel correlates the pseudo colors and the slow axis orientation. cc, Claudius cells; cd Deiters Cells; he, Hensen cells; ih, inner hair cell; ip, inner pillar cell; oh, outer hair cell; op, outer pillar cell; tc, tunnel of Corti.

Polarized light microscopy^33–37^ was used to measure the birefringence of fine structures in the BM to obtain detailed analyses of changes in the anisotropy of the BM during its development (Supplementary Fig. 1). Birefringence imaging was performed on thin optical sections in 0.5 μm increments of acutely isolated, unfixed BM, which avoided artifacts associated with fixation and dehydration (Supplementary Fig. 2). The technique examines the anisotropy of the refractive index in birefringent materials using polarized of light. The collagen fibers of the BM are intrinsically birefringent^38–41^ and constitute its dominant birefringent component. The fiber orientation in the thin optical sections is revealed by the axis of high refractive index, also called slow axis^33,38^. The slow axis or fiber orientation is shown in pseudocolor images with a color wheel that associates a specific hue with an orientation (Fig. 1G). Our data confirm that the collagen networks of the BM are positive birefringent materials (Fig. 1B,D) where the extraordinary refractive index is greater than the ordinary index and the slow optic axis is oriented along the axis of the fiber, consistent with previous reports (Fig. 1C,E;^38,40^). To study structural differences in the developing BM architecture, images were collected at various stages of development along the tonotopic axis. Example of images collected from the basal end of immature (P10) and mature (P20) BM in Figs. 1C and E show developmental differences in the arrangement and orientation of fibers as reflected in the greater uniformity in color of the mature BM.

### Developmental changes in BM structure

**Figure 2:**
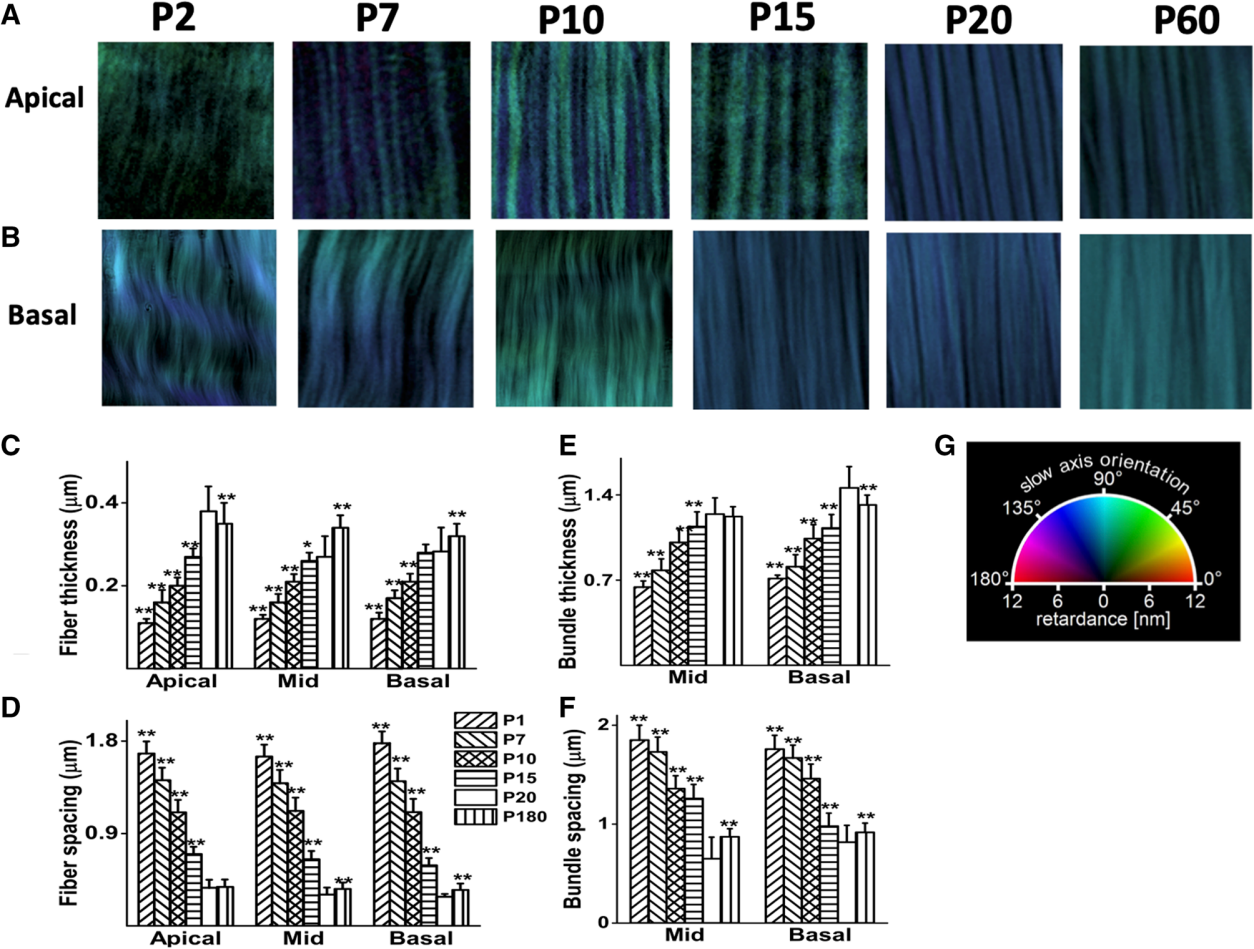
Changes in BM structure at different stages of development. (**A**) Examples of LC-PolScope images of collagenous fibers in unfixed BM preparations at P2, P7, P10, P15, P20 and P60 from apical turns of the cochlea corresponding to the ~ 7 kHz region, corresponding to location about 90% of BM length, with base being 0% and apex 100%^42^. Sample size shown is 10 × 10 μm. (**B**) Examples of LC-PolScope images of collagenous fibers in unfixed BM preparations at P2, P7, P10, P15, P20 and P60 from basal turns of the cochlea corresponding to the ~ 60 kHz region, corresponding to location about 10% of BM length, with base being 0% and apex 100%^42^. Sample size shown 10 × 10 μm. (**C**) Fiber thickness in apical, midturn and basal regions of BM all increase significantly (as indicated by asterisks, see below) in size with age to P20 (as indicated by different column hatchings, see key for postnatal ages P1 to P60), but not significantly between P15 and P20 in cochlear basal turn. Fiber thickness continues to increase with age to P60 in mid and basal turns and decreases in apical turns. (**D**) Fiber spacing decreases with postnatal age to P20 in the BM apical, midturn and basal regions and increases significantly between P20 and P60 in the mid and basal turns. (**E**) Bundle thickness increases with age to P20 in the BM midturn and basal regions. Bundles were not observed in apical turns. Bundle thickness decreases significantly between P20 and P60 in the basal turn. (**F**) Bundle spacing in the BM midturn and basal regions decreases with age to P20 and increases from P20 to P60. (**G**) Color wheel shows a visual vectorial representation of orientations. A color wheel correlates the pseudo colors and the optical axis orientation. The reference (zero degrees) is the longitudinal axis of basilar membrane or X axis when you align the images of fibers along Y axis. Similar fiber colors indicate uniform alignment; color variations indicate nonuniformity in alignment. The measurements used for (**C**)–(**F**) were derived from measuring 200 samples for each age and location, using BM samples from six cochleae (3 mice) per age group, means ± SD. , *p < 0.001 and **p < 0.0001, two-tailed unpaired *t* test comparing measurements taken at different ages with P20.

To better understand the dynamics of BM structural development in the pectinate zone of the BM (Fig. 1A), a detailed analysis of BM structural organization was performed from the day of birth (P1) until 6 months old, where P20 is the time of hearing onset in mice (Fig. 2). The arbitrary orientation of BM sections in each preparation resulted in color differences between preparations (Fig. 2A,B). To facilitate the comparions between samples, image X axis was used as zero degree for the fibers that are aligned along Y axis. Within a given preparation, the slow axis orientation, as reflected in the pseudocolor coded presentations of optical axis of orientation, varied along the tonotopic axis for the preparations < P15, indicating disarrangement of fibers in the early stages of postnatal development (Fig. 2A,B showing apical and basal ends for comparison). The colors became more uniform in preparations over ~ 10 days of age, indicating that the fiber orientation was more uniform at older stages (Fig. 2A,B). In the apical region, only fibers could be observed, while fiber bundles were dominant in the more basal regions. These observations are consistent with the relatively sparse presence of collagenous filaments in apical compared to mid-basal regions of the BM, as shown by electron microscopy^9,10^.

Moreover, in the apical region, fiber thickness increased progressively and spacing of fibers reduced with development (Fig. 2C, D), reaching maturity around P20. A similar trend was observed in more basal regions of the BM, where bundles of fibers became thicker and more closely packed with maturation, reaching a mature form by P20 (Fig. 2E,F).

**Figure 3:**
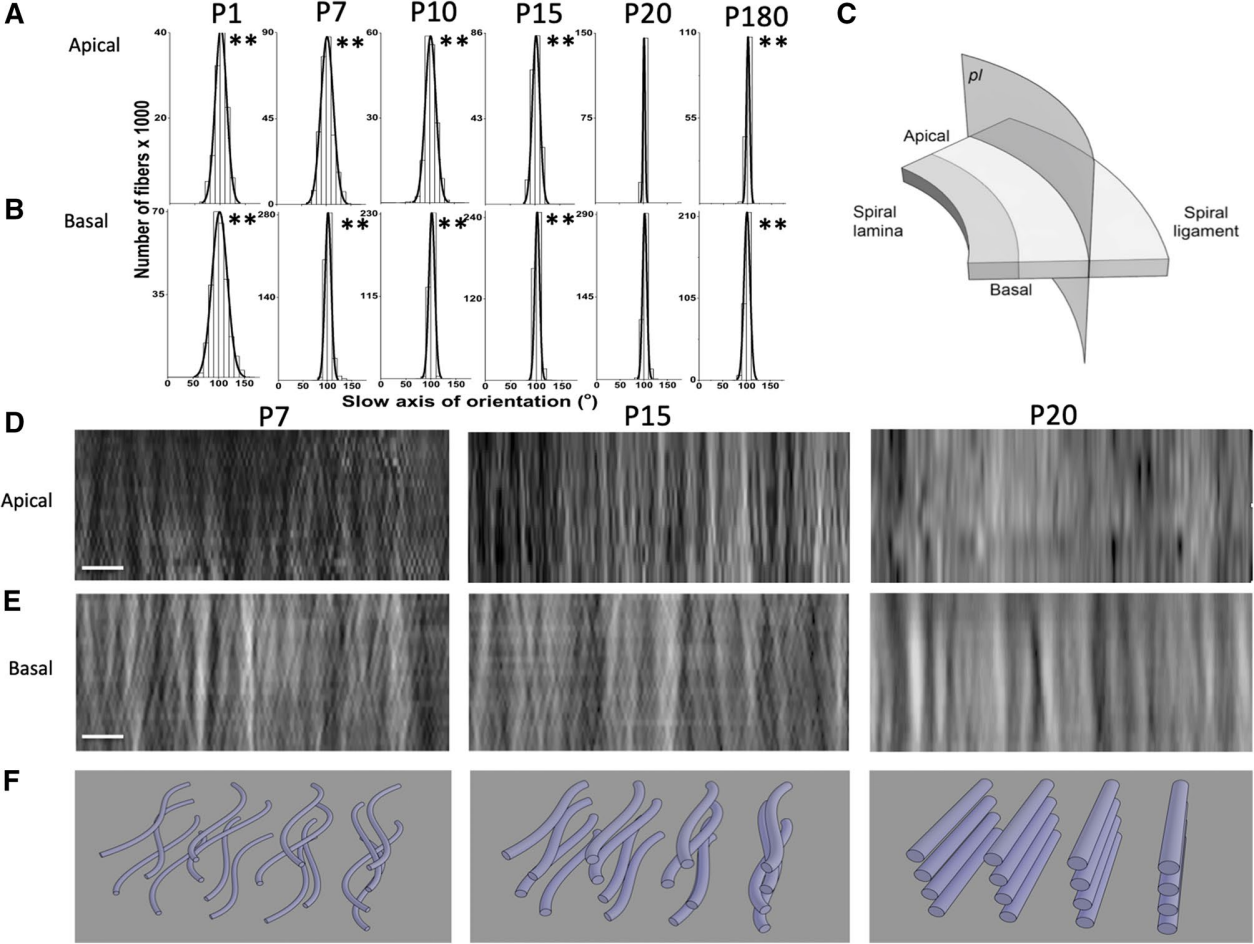
BM fibers become more narrowly orientated with increased alignment and decreased twisting with advancing development. (**A**, **B**) Histograms of BM fiber orientation as function of developmental stage (P1 to P180 for measurements in the apical (**A**) and basal (**B**) cochlea regions. Fiber orientations were assigned to 10° bins. The mean orientation in each preparation was normalized (to ~ 101°) and the resultant histograms were fitted with normal distribution curves. Table 1 lists SD from the mean. Groups, three mice (six cochleae); differences in SD, **p < 0.0001, *F* test statistics. Fiber orientations for the apical turn were measured from optical sections (0.5 μm apart in 8 optical sections). Measurement numbers for each developmental age: P1 n = 35,693 ; P7 n = 17,064 ; P10 n = 90,001; P15 n = 193,020 ; P20 n = 163,805 ; P180 n = 166,203, using BM samples from six cochleae (3 mice) per age group. Fiber orientations measured for the basal turn were measured from optical sections (0.5 μm apart in 20 optical sections). Measurement numbers for each developmental age: P1 n = 261,740; P7 n = 417,994; P10 n = 283,742; P15 n = 365,359; P20 n = 145,339; P180 n = 147,231, using BM samples from six cochleae (3 mice) per age group. (**C**, **D**) Birefringent images constructed from optical stacks of transverse sections taken through the pectinate zone of the BM have a more homogeneous and less braded appearance with increasing stages of development. (**C**) Schematic diagram illustrating the plane of optical sections represented in (**D** and **E**). (**D**) Transverse (plane *pl* in **C**) filament array images reconstructed from apical stacks of pectinate zone optical sections for P2, P7, P10, P15, P20 and P60 (0.5 μm apart; 8 apical and 18 middle optical sections; total thicknesses 4 μm, respectively). Scale bar 1 μm. E. Transverse (plane *pl* in **C**) filament array images reconstructed from basal stacks of pectinate zone optical sections for P2, P7, P10, P15, P20 and P60 (0.5 μm apart; 18 basal optical sections; total thicknesses 9 μm, respectively). Scale bar 5 μm. (**F**) Schematic diagrams of the alignment and curvature of the BM fibers at P7, P15 and P20 that would account for the increased homogeneity in birefringence and decreased degree of braiding with advancing development seen in the optical sections shown in C and D. See Supplementary Fig. 3 for a video animation.

To characterize the BM structural signature in more detail, optical sections at 0.5 μm intervals were collected to track the distribution and arrangement of fibers throughout the depth of the BM structure (Supplementary Fig. 2) and the axis of orientation was measured to quantify the arrangement of collagenous elements within the preparation (Fig. 3). The histograms were taken at 10-degree intervals, ranging from 0 to 180 degrees and the standard deviation (SD) of the measurements was used to indicate the disorganization of the collagenous elements (Methods- statistical analysis, and Fig. 3A,B). There were statistically significant differences in fiber orientation at all stages of development compared to P20, taken as the adult form, the age of onset of hearing in mice. The broader distribution of fiber orientations in the younger and aging animals is reflected in the larger SD measured (Table 1).

**Table 1:** Differences between slow axis of orientation in developing BM.

	P2	F	*p*	P7	F	*p*	P10	F	*p*	P15	F	*p*	P20	P180	F	*p*
Apical	15.08	0.02	< 0.0001	10.98	0.04	< 0.0001	10.12	0.05	< 0.0001	7.67	0.08	< 0.0001	2.20	3.27	0.45	< 0.0001
Basal	11.43	0.17	< 0.0001	8.90	0.27	< 0.0001	6.24	0.06	< 0.0001	5.91	0.57	< 0.0001	4.70	6.35	0.55	< 0.0001

Slow axis of orientation was measured in the apical and basal regions at different stages of development. SD were compared, as a measure of fiber disarrangement. The developmental disorganization of the collagenous elements as reflected in the SD. The comparison between different ages versus P20 was made, as reflected in reported F and *p* values.

Differences in structural alignment of the developing BM and quantification of how the fibers were extended or/and twisted (Fig. 3D,E) was determined by tracking the optical axis orientations of the fibers through the BM volume (in longitudinal transverse views through the pectinate zone , i.e. a cross-section defined in Fig. 3C). The fibers are more twisted and braided earlier in development (< P15, Fig. 3. However, as the BM matures, the fibers become more aligned. The images shown in Fig. 3D,E represent a unique structure of extracellular matrix (ECM) in the BM which, in its mature form, looks like an alignment of flat sheets of extracellular matrix that run perpendicular to the length of the BM. A schematic representation summarizing the main observations of BM structural development is shown in Fig. 3F and Supplementary movie.

Together, these results provided evidence that the collagenous fibers become more compact and tightly aligned as development progressed, and that the alignment of fibers was preserved through the length of the BM^43^. This is the first study which demonstrates the developmental changes in 3D BM structure. The mature BM form resembles a structure analogous to that of an airplane wing, composed of many vertical ribs that align perpendicular the length of the wing, which give some flexibility for the deflection of the wing while maintaining mechanical strength (Fig. 3F, Supplementary movie).

### Role of emilin2 in maturation of BM

**Figure 4:**
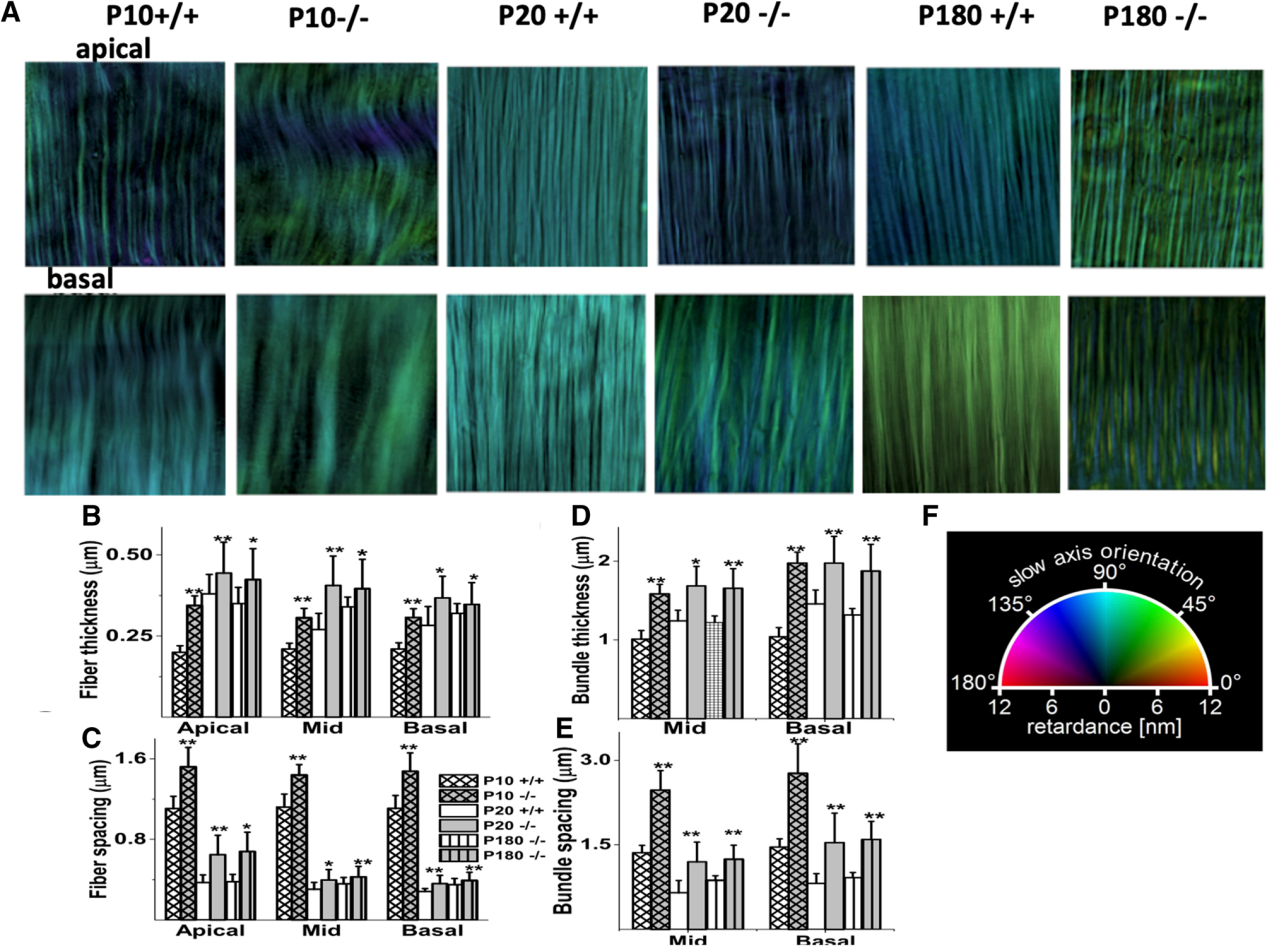
Changes in BM structure at different stages of development between wild type and *Emilin2*^−/−^ mice. (**A**) Examples of LC-PolScope images of collagenous fibers in unfixed BM preparations at P10, P20 and P60 from the wild type and emi-2^−/−^ cochlea. Top row corresponds to the ~ 7 kHz apical region, The bottom row corresponds to the ~ 60 kHz basal region Sample size shown is 10 × 10 μm. Similar fiber colors indicate uniform alignment; varied colors indicate nonuniformity. (**B**) Fiber thickness in apical, midturn and basal regions of -/- BM all increase significantly (as indicated by asterisks, see below) in size with age compared to emilin^+/+^ BM (as indicated by different column hatchings, see key for postnatal ages P1, P20, P180). (**C**) Fiber spacing is significantly higher in in apical, midturn and basal regions in emilin^-/-^ compared to emilin^+/+^ BM. (**D**) Bundle thickness in apical, midturn and basal regions of emilin^-/-^ BM all increase significantly in size with age compared to + / + BM. Bundles were not observed in apical turns. (**E**) Bundle spacing is significantly higher in in apical, midturn and basal regions in emilin^-/-^ compared to emilin^+/+^ BM. (**F**) Color wheel shows a visual vectorial representation of orientations. A color wheel correlates the pseudo colors and the optical axis orientation. The reference (zero degrees) is the longitudinal axis of basilar membrane or X axis when you align the images of fibers along Y axis. Similar fiber colors indicate uniform alignment; color variations indicate nonuniformity in alignment. The measurements used for (**C**)–(**F**) were derived from measuring 200 samples for each age and location, using BM samples from six cochleae (3 mice) per age group, means ± SD. , *p < 0.001 and **p < 0.0001, two-tailed unpaired *t* test comparing measurements taken at different ages with P20.

Emilin2 was recently reported to be responsible for providing the mature BM with elasticity and mechanical frequency resolution^18^. We, therefore, investigated the developmental role of emilin2 in BM maturation. The BMs of mice that do not express emilin2 (emilin2^−/−^) appear to display an immature phenotype throughout life (Fig. 4A), resulting in reported functional consequences (Russell et al., 2020). Within a given emilin2 ^+/+^ preparation, the image color was constant for > P10, indicating uniform alignment of collagenous fibers within the preparation, (Fig. 4A top row). In contrast, in emilin2^−/−^ mice, this uniformity was disturbed, as indicated by the varied colors of fibers within a preparation throughout development and maturation (Fig. 4A bottom row). The thickness and spacing of fibers and bundles of fibers also showed increases in emilin2^−/−^ compared to emilin2^+/+^ preparations, consistent with the disorder of these structures (Fig. 4B–E).

**Figure 5:**
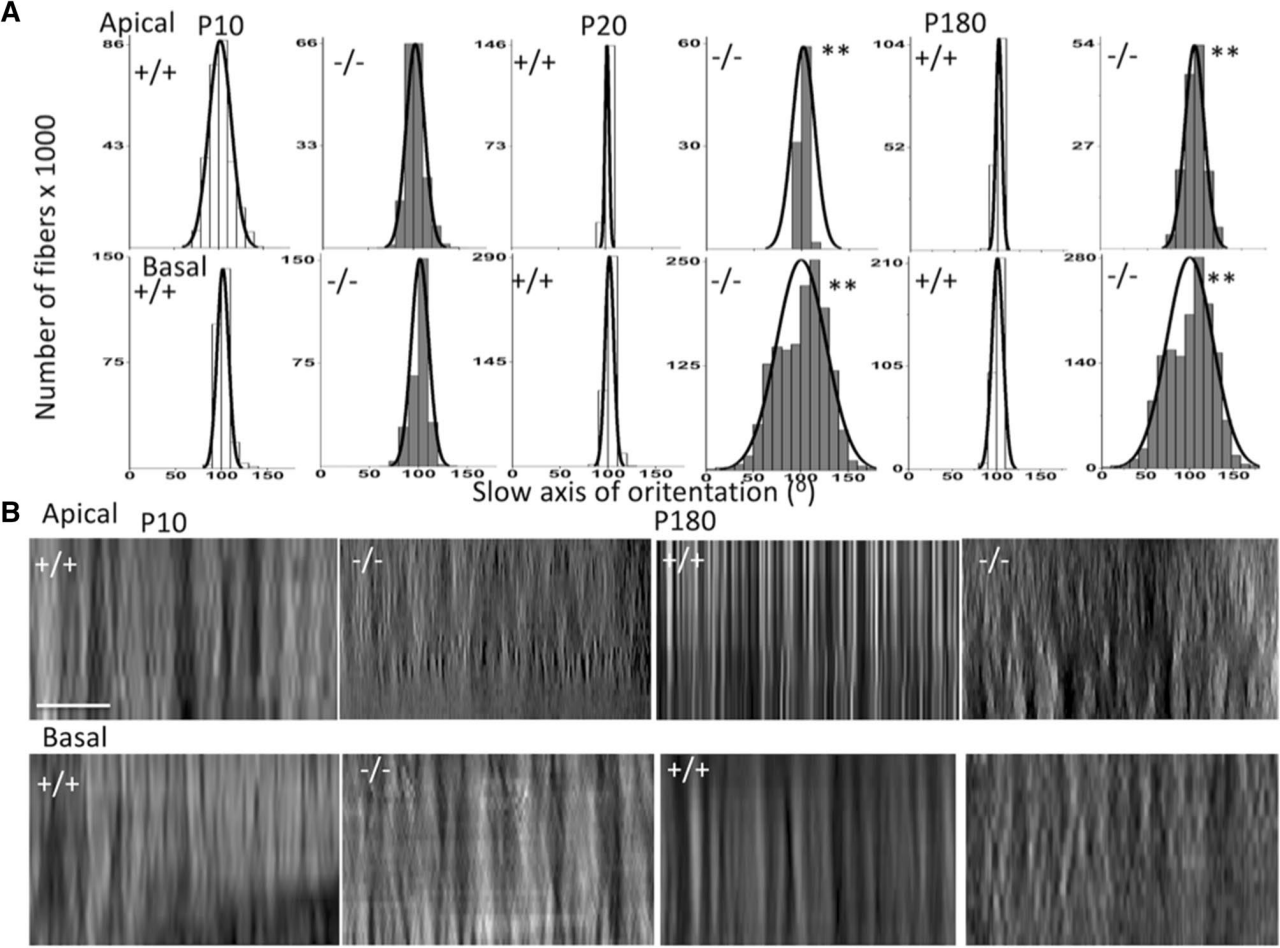
BM fibers become more misaligned and twisted in *Emilin2*^−/−^ mice to compared to wild type BM. (**A**) Histograms of BM fiber orientation as function of developmental stage (P10, P20 to P180) for measurements in the apical (top row) and basal (bottom row) cochlea regions. Fiber orientations were assigned to 10° bins. The mean orientation in each preparation was normalized (to ~ 101°) and the resultant histograms were fitted with normal distribution curves. Table 2 lists SD from the mean. Groups, three mice (six cochleae); differences in SD, **p < 0.0001, *F* test statistics. Fiber orientations for the apical turn were measured from optical Sects. (0.5 μm apart in 8 optical sections). Measurement numbers for each developmental age: P10 n = 90,001; P10^-/-^ n = 53,920 ; P20 n = 163,805 ; P20^-/-^ n = 164,805;P 180 n = 166,203; P180 ^-/-^ n = 167,203 ; using BM samples from six cochleae (3 mice) per age group. Fiber orientations measured for the basal turn(bottom row) were measured from optical Sects. (0.5 μm apart in 20 optical sections). Measurement numbers for each developmental age: P10 n = 283,742; P10^-/-^ n = 27,246; P20 n = 145,339; P20^-/-^ n = 151,419; P180 n = 147,231, P180 ^-/-^ n = 149,250 using BM samples from six cochleae (3 mice) per age group. (**B**) Birefringent images constructed from optical stacks of transverse sections taken through the pectinate zone of the BM have less homogeneous and more braded appearance with increasing stages of development in emilin^-/-^ compared to emilin^+/+^ BM. Transversal (plane *pl* in Fig. 3E) filament array images reconstructed from stacks of pectinate zone optical sections for P10, P20 and P60 emilin^+/+^ and emilin^−/−^ cochleae (0.5 μm apart; 8 apical and 18 middle optical sections; total thicknesses are 4 and 9 μm, respectively). Scale bar 5 μm.

The histograms in Fig. 5A depict a wider range of orientations measured in emilin2^−/−^ than emilin2^+/+^ preparations in apical and basal turns of the cochlea. The wider distribution of fiber orientations in emilin2^−/−^ mice is indicated by the considerably greater SDs of the data (Table 2). The transversal cross sections of emilin2^-/-^ BM (Fig. 5 B) confirm the disarrangement of fibers, as recently reported (Russell et al. 2020). Stacks of optical sections were used to reconstruct the filamentous array of the BM in longitudinal transverse views through the pectinate zone (Fig. 5B). In emilin2^+/+^ mice, the reconstruction showed vertically oriented fiber bundles. This pattern can occur if the radial bundles are arranged immediately above each other, similar to the stringer supports inside an aircraft wing. In emilin2^−/−^ mice, the pattern appeared warped or braided with an offset arrangement of bundles, which was more apparent in thicker, mid-basal turns than in thinner apical turns of the BM.

**Table 2:** Differences between slow axis of orientation in wild type and *Emilin2*^−/−^ BM.

	P10 + +	P10/-	F	*p*	P20 + / +	p20/-	F	*p*	P180 + / +	P180/-	F
Apical	10.12	12.69	0.64	0.89	2.20	11.89	0.03	< 0.0001	3.27	12.2	0.07
Basal	6.24	6.84	0.83	0.82	4.70	28.07	0.03	< 0.0001	6.35	27.25	0.05

Slow axis of orientation was measured in the apical and basal regions of emilin^+/+^ and emilin^−/−^ BM and SD were compared, as a measure of fiber disarrangement. The disorganization of the collagenous elements^−/−^ BM as reflected in SD. The comparison between different ages in emilin^+/+^ and emilin^−/−^ were made, as reflected in reported F and *p* values.

## Discussion

This study is the first to provide direct evidence of the 3D structural development and maturation of mammalian BM, which was hypothesized to underlie the developmental maturation of tonotopic maps and frequency tuning in animal models^22,23,26,28,44–46^ as well as in newborn humans^47^. The results of this study show an increase in fiber thickness and more compact ordering with maturation (Figs. 1 and 2). The fibers in the early period of maturation (*p* < 15) show a wavier pattern, however, they seem to be aligned throughout the BM, without the braiding pattern observed in the emilin2^−/−^ (Figs. 3 and 5, Supplementary Fig. 3). Moreover, there is a temporal delay in maturation, with the apical cochlear region lagging behind the basal region, consistent with the general maturation of the OC (reviewed in^48^). Thus, BM structural maturation is temporally coupled to the maturation of hearing (reviewed in^49^).

One of the possible molecular pathways guiding the structural maturation of the BM seems to involve emilin2, expression of which starts around the time of birth, peaks around P15 (17) and is observed throughout the life span, suggesting that it is a constitutive member of the BM. Interestingly, the emilin2 ^-/-^ BM shows a more braided pattern of fibers than in the control animals, even at P10, when lower levels of emilin2 are detected in the BM (^17^, Fig. 5B), compared to more mature stages (P20, P180), when the phenotype is also more pronounced (Fig. 5).

Our study focused on development (up to 1 month old), and included animals 6 month old, when the onset of age related age hearing loss has been reported. Namely, studies investigating age related hearing loss in C57B/J animals using the auditory brainstem response (ABR) test, showed the hearing threshold levels of the mice increased progressively starting at about 6 months of age, significantly worsening by 15 months of age^50–52^. Moreover, the observed decrease in the characteristic frequency would be expected with decline of the aging cochlear amplifier but decline in the amplification even in C57B/J mice is, probably, a complex process (i.e. not only changes in the BM morphology which still can contribute though) with contribution from strial atrophy and EP decline^53^.

Therefore, the structural differences observed during development and at 6 months old can be attributed to the emilin-2 protein. Moreover, the fact that we observe also structural alterations in control animals between 21 days and 6 month old animals suggests that potentially age related hearing loss may be also reflected in the structural changes in basilar membrane structure, although the causal relationship is yet to be established. Therefore, we expect that the Ahl gene, associated with age related hearing loss, has no effect on BM structure during development, while it could be involved in the alterations in BM structure observed at 6 months old mice^54,55^ However, since both Emilin − 2^−/−^ and Emilin − 2^+/+^ animals were on the same genetic background, we feel confident that any structural differences we report are due to alterations in emilin2 protein.

Therefore, it is safe to conclude that emilin2 signaling contributes to the fine structural maturation of the BM throughout development, in addition to maturation^18^. The present findings also suggest that in the emilin2^−/−^ mutant BM development is arrested, as maturation does not proceed as observed in the BM of wild-type mice.

The current findings provide evidence for structural changes in the BM with age, consistent with the reports indicating developmental changes in BM collagen expression^12^. We are confident that the fibers observed are collagen, based on the published immunohistochemistry and biochemical evidence^56^. Namely, the most abundant constituent of the basilar membrane are collagen fibers which are also regarded as a main source of basilar membrane stiffness, thereby playing a critical role in establishing its tuned resonant frequency map^9,57,58^. They are also highly birefringent as shown in other studies^38–40,59^. Thus coupled together, we feel confident to presume that the fibers being examined are indeed collagen fibers, as no other structural element is known to meet the above criteria. However, it is not excluded that we were imaging some other biochemically unidentified elements, although if they were so abundant in the BM, one would expect that they would have been identified readily so far. Compared to mature BM at P20, collagenous fibers arrangement may undergo further changes in aging animals (Fig. 2,^60^) Thus, it is likely that the BM is a dynamic structure which changes throughout the life span of the animal, which is consistent with reported age-related changes in human BM^61^. The importance of BM structural contribution to normal hearing is highlighted in human patients with mutations in BM structural collagen proteins. For example, Stickler syndrome, resulting in variants in coding Collagen II or IX,^62^; or Alport syndrome, resulting in pathogenic variants in Collagen IV^63^ can lead to sensorineural hearing loss in humans. Therefore, it is conceivable that BM structure may reflect the underlying changes in hearing, such as in noise induced hearing loss (NIHL) or age-related hearing loss (presbycusis). While structural changes associated with NIHL or presbycusis are yet to be reported, there is other evidence to suggest BM involvement in pathological states^64^. For instance, cochlear implantation induced collagen deposition, resulting in BM stiffening in the basal turn of the guinea pig cochlea^65^.

In addition to providing an anatomical basis for tonotopic maps and frequency tuning, the BM may interact with the cellular responses of the OC sensory and supporting cells, with which it in close contact, thereby further influencing cochlear responses beyond alterations in the tonotopicity of the BM^66,67^. Macrophages located within the BM activate in response to sensory cell damage during acute stress (e.g. NIHL) and chronic stress (e.g. presbycusis). Macrophages are the major innate immune cells in the cochlea, and important drivers of inflammatory and tissue repair responses after cochlear injury^68–70^. However, it is not clear if the sensory cells become damaged first and then initiate a macrophage immune response, or if perhaps there is dysregulation of cochlear immune responses within the BM which cause damage to the sensory epithelium^71^. Either way, this is one example which illustrates the possibility that structural elements of the BM interact with their environment, including the sensory epithelium. This also suggests that the BM is capable of adaptation to changes in the local sensory epithelium environment influenced by sensory cell status. Moreover, the changes in BM responses and passive mechanics were reported in cochlear connexin30 A88V mutants^72^. Connexin 30 is one of two most prevalent gap junctional proteins localized in the cochlear supporting cells which are located on top of the BM. This creates a possibility for a structural remodeling of the BM, due to signaling alterations in supporting cells, which is yet to be investigated^73^.

The BM is an example of an ECM, which is a highly dynamic, three-dimensional acellular structure that is present in all tissues and is essential for life^74^. The ECM is shown to provide structural support, cell adhesion and to play a vital role in the determination, differentiation, proliferation, survival, polarity, and migration of cells in many tissues, including central nervous system^75^. Therefore, the ECM signals generated by the BM may be important in guiding the normal development and functioning of the OC and are likely to be involved in regulating development and cellular differentiation and signaling in the OC throughout the life of the animal^76^.

BM provides structural environment that could determine mechanical properties of the cells within the sensory epithelium. Moreover, the immediate mechanical and electrical environment might have a significant role in controlling the maturation and frequency-dependent properties of the cellular elements of the OC. There are a few studies which demonstrate that maturation of IHCs and their efferent innervation depend on mechanotransduction^77,78^. Because mechanotransduction depends on the immediate mechanoelectrical environment of HCs, including properties of the developing BM, it is sensible to suggest that developing local mechanical properties and tonotopy of the BM is an important part of the cochlear maturation. There is also an evidence that mechanical forces drive ordered patterning of hair cells in the mammalian inner ear and changes in the BM mechanical properties must be a crucial player here^79^. Thus, it may not be a pure coincidence that the tonotopicity of cochlear cellular elements and parallels that of the BM^41,80–82^. Moreover, the tonotopicity is probably not entirely governed through genetics but is also influenced by the local electromechanical environment. For example, the maturation of mechanoelectrical transduction in IHCs is determined by experiencing mechanical stimulation^77^.

Therefore, it is imperative to fully understand the composition of BM, how it changes with development, maturation and different pathological states along the tonotopic axis and to explore the only partially understood roles of the BM and ECM. Together, this information will further our understanding of ECM-cell interactions mediating hearing functions, which is of utmost importance for understanding the structural contributions BM plays in the OC development and the transduction of the auditory signals. A better understanding of how the BM and ECM regulates organ structure and function and how the BM and ECM remodeling affects auditory processing will potentially contribute to the development of new therapeutics^63^.

## Materials and methods

### Experimental methods

#### *The methods are similar to the ones used by Russell *et al. *2020*^18^

*Tissue isolation*: Mice were housed under standard conditions with 12-h light/12-h dark cycles with food and water ad libitum. Wild type C57B/J mice aged from P1- 6 month old were used for experiments. The animals were killed by cervical dislocation, the temporal bones were removed and the inner ear harvested and stored in ice cold Leibovitz's medium (L-15 Medium, Thermo Fisher Scientific, catalogue number 21083). The cochleae were isolated and basilar membranes dissected in ice cold Leibovitz's medium. Samples of apical, middle, and basal turns of the cochlea were separated. Each sample was then examined in a drop of the bath solution, under a coverslip, using a Nikon microscope with an oil immersion 60x/0.9NA objective lens and equipped with a QImaging Retiga 2000R camera with resolution of 0.1075 μm/pixel. The cochlear samples from Emilin2^−/−^ mice were obtained from Dr Forrest’s lab at National Institute of Diabetes and Digestive and Kidney Diseases (NIDDK). Temporal bones were isolated from mice at NIDDK and shipped overnight to the Marine Biological Laboratory (MBL; Woods Hole, MA). Samples were shipped in ice-cold Leibovitz’s medium and arrived at MBL within ~ 18 h of harvesting. No difference was observed whether control samples were shipped from NIDDK or were isolated at MBL, suggesting that shipment of material did not change outcomes. At MBL, cochleae were isolated and the BM was dissected in ice-cold Leibovitz’s medium. The tissue was processed as described above. *Ethics declaration*: Studies were performed in accordance with approved protocols at NIDDK at the NIH and approval from the MBL, Woods Hole, Animal Welfare and Ethical Review Body. The authors complied with the ARRIVE guidelines.

*Birefringence imaging*: LC-PolScope and Polychromatic PolScope were used to measure magnitude (retardance) and orientation (slow optical axis) of BM birefringence^18^ Fiber orientations were determined using a color wheel. Images were analyzed using OpenPolScope software (www.openpolscope.org), which included Micro-Manager version 1.4.22 and OpenPolScope version 3.2, with further analysis using Fiji (ImageJ), Photoshop (Adobe), and Origin software (MicroCal LLC, www.originlab.com). Samples were imaged blind, without knowledge of genotypes, which were confirmed later.

### Statistical analysis

Where appropriate, pooled data are presented as mean ± S.D. Significant differences between the control and the experimental groups were tested using Student’s unpaired *t*-test (*p < 0.001, **p < 0.0001) using Origin software. To test whether the orientation of fibers was significantly different between genotypes, histograms of fiber orientation lines were plotted where measured angles were assigned to 18 bins, each representing 10° of orientation. To compare preparations with varied absolute orientations, the mean orientation of each preparation was normalized (to ~ 101°) by shifting orientation lines (without altering distribution patterns). Standard Deviation (SD) was used to calculate the coefficient of variance and then performed an *F* test using Origin software to test whether data samples came from populations with equal variances. Corresponding *p* values were determined from *F* test values using Origin. The thickness of fibers and fiber bundles was measured on images obtained from Photoshop (Adobe) and then analyzed using Origin software.

## Supplementary Information

Supplementary Information 1.

Supplementary Video 1.

## Data Availability

All data is available in the main text or the supplementary materials. Additional data related to this paper may be requested from the authors.

## References

[1] Bekesy G (1960). Experiments in Hearing.

[2] Dallos P (1975). Electrical correlates of mechanical events in the cochlea. Audiol. Off. Organ Int. Soc. Audiol..

[3] Robles L, Ruggero MA (2001). Mechanics of the mammalian cochlea. Physiol. Rev..

[4] Sumner CJ, Wells TT, Bergevin C, Sollini J, Kreft HA, Palmer AR, Oxenham AJ, Shera CA (2018). Mammalian behavior and physiology converge to confirm sharper cochlear tuning in humans. Proc. Natl. Acad. Sci. U.S.A..

[5] Nuttall AL, Ricci AJ, Burwood G, Harte JM, Stenfelt S, Cayé-Thomasen P, Ren T, Ramamoorthy S, Zhang Y, Wilson T, Lunner T, Moore BCJ, Fridberger A (2018). A mechanoelectrical mechanism for detection of sound envelopes in the hearing organ. Nat. Commun..

[6] Slepecky N, Dallos P, Popper A, Fay R (1996). Structure of the mammalian cochlea. The Cochlea.

[7] Goycoolea MV, Muchow D, Schachern P (1988). Experimental studies on round window structure: function and permeability. Laryngoscope.

[8] Keithley EM, Ryan AF, Woolf NK (1993). Fibronectin-like immunoreactivity of the basilar membrane of young and aged rats. J. Comp. Neurol..

[9] Iurato S (1962). Submicroscopic structure of the membranous labyrinth. III. The supporting structure of Corti’s organ (basilar membrane, limbus spiralis and spiral ligament). Z. Zellforsch. Mikrosk. Anat..

[10] Cabezudo LM (1978). The ultrastructure of the basilar membrane in the cat. Otolaryngology.

[11] Katori Y, Hozawa K, Kikuchi T, Tonosaki A, Takasaka T (1993). Fine structure of the lamina basilaris of guinea pig cochlea. Acta Otolaryngol..

[12] Cosgrove D, Kornak JM, Samuelson G (1996). Expression of basement membrane type IV collagen chains during postnatal development in the murine cochlea. Hear. Res..

[13] Cosgrove D, Samuelson G, Pinnt J (1996). Immunohistochemical localization of basement membrane collagens and associated proteins in the murine cochlea. Hear. Res..

[14] Dreiling FJ, Henson MM, Henson OW (2002). The presence and arrangement of type II collagen in the basilar membrane. Hear. Res..

[15] Thalmann I (1993). Collagen of accessory structures of organ of Corti. Connect. Tissue Res..

[16] Tsuprun V, Santi P (1999). Ultrastructure and immunohistochemical identification of the extracellular matrix of the chinchilla cochlea. Hear. Res..

[17] Amma LL, Goodyear R, Faris JS, Jones I, Ng L, Richardson G, Forrest D (2003). An emilin family extracellular matrix protein identified in the cochlear basilar membrane. Mol. Cell. Neurosci..

[18] Russell IJ, Lukashkina VA, Levic S, Cho YW, Lukashkin AN, Ng L, Forrest D (2020). Emilin 2 promotes the mechanical gradient of the cochlear basilar membrane and resolution of frequencies in sound. Sci. Adv..

[19] Harris DM, Dallos P (1984). Ontogenetic changes in frequency mapping of a mammalian ear. Science.

[20] Yancey C, Dallos P (1985). Ontogenic changes in cochlear characteristic frequency at a basal turn location as reflected in the summating potential. Hear Res..

[21] Arjmand E, Harris D, Dallos P (1988). Developmental changes in frequency mapping of the gerbil cochlea: comparison of two cochlear locations. Hear Res..

[22] Echteler SM, Armjand E, Dallos P (1989). Developmental alterations in the frequency map of the mammalian cochlea. Nature.

[23] Müller M (1991). Developmental changes of frequency representation in the rat cochlea. Hear. Res..

[24] Lippe W, Rubel EW (1983). Development of the place principle: tonotopic organization. Science.

[25] Rubel EW, Ryals BM (1983). Development of the place principle: acoustic trauma. Science.

[26] Overstreet EH, Temchin AN, Ruggero MA (2006). Passive basilar membrane vibrations in gerbil neonates: mechanical bases of cochlear maturation. J. Physiol..

[27] Emadi G, Richter CP (2008). Developmental changes of mechanics measured in the gerbil cochlea. J. Assoc. Res. Otolaryngol. JARO.

[28] Schweitzer L, Lutz C, Hobbs M, Weaver SP (1996). Anatomical correlates of the passive properties underlying the developmental shift in the frequency map of the mammalian cochlea. Hear Res..

[29] Munyer PD, Schulte BA (1995). Developmental expression of proteoglycans in the tectorial and basilar membrane of the gerbil cochlea. Hear Res..

[30] Kuhn B, Vater M (1997). The postnatal development of F-actin in tension fibroblasts of the spiral ligament of the gerbil cochlea. Hear Res..

[31] Souter M, Nevill G, Forge A (1997). Postnatal maturation of the organ of Corti in gerbils: morphology and physiological responses. J. Comp. Neurol..

[32] Koike-Tani M, Tani T, Mehta SB, Verma A, Oldenbourg R (2015). Polarized light microscopy in reproductive and developmental biology. Mol. Reprod. Dev..

[33] Oldenbourg R, Salmon ED, Tran PT (1998). Birefringence of single and bundled microtubules. Biophys. J ..

[34] Shribak M, Oldenbourg R (2003). Techniques for fast and sensitive measurements of two-dimensional birefringence distributions. Appl. Opt..

[35] Shribak M (2015). Polychromatic polarization microscope: bringing colors to a colorless world. Nat. Sci. Rep..

[36] Inoue S (1953). Polarization optical studies of the mitotic spindle. I. The demonstration of spindle fibers in living cells. Chromosoma.

[37] Inoue S, Hyde WL (1957). Studies on depolarization of light at microscope lens surfaces. II. The simultaneous realization of high resolution and high sensitivity with the polarizing microscope. J. Biophys. Biochem. Cytol..

[38] Petroll WM (2006). Differential interference contrast and confocal reflectance imaging of collagen organization in three-dimensional matrices. Scanning.

[39] Spiesz EM, Kaminsky W, Zysset PK (2011). A quantitative collagen fibers orientation assessment using birefringence measurements: calibration and application to human osteons. J. Struct. Biol..

[40] Kalwani NM, Ong CA, Lysaght AC, Haward SJ, McKinley GH, Stankovic KM (2013). Quantitative polarized light microscopy of unstained mammalian cochlear sections. J. Biomed. Opt..

[41] Beurg M, Cui R, Goldring AC, Ebrahim S, Fettiplace R, Kachar B (2018). Variable number of TMC1-dependent mechanotransducer channels underlie tonotopic conductance gradients in the cochlea. Nat. Commun..

[42] Müller M, von Hünerbein K, Hoidis S, Smolders JW (2005). A physiological place-frequency map of the cochlea in the CBA/J mouse. Hear Res..

[43] Mikuni H, Fukuda S, Küçük B, Inuyama Y, Ushiki T, Abe K (1995). The three-dimensional fibrillar arrangement of the basilar membrane in the mouse cochlea. Eur. Arch. Oto-Rhino-Laryngol..

[44] Romand R (1997). Modification of tonotopic representation in the auditory system during development. Prog. Neurobiol..

[45] Kössl M, Foeller E, Drexl M, Vater M, Mora E, Coro F, Russell IJ (2003). Postnatal development of cochlear function in the mustached bat, Pteronotus parnellii. J. Neurophysiol..

[46] Emadi G, Richter CP (2008). Developmental changes of mechanics measured in the gerbil cochlea. J. Assoc. Res. Otolaryngol. (JARO).

[47] Meenderink S, Shera CA, Valero MD, Liberman MC, Abdala C (2019). Morphological immaturity of the neonatal organ of corti and associated structures in humans. J. Assoc. Res. Otolaryngol. JARO.

[48] Basch ML, Brown RM, Jen HI, Groves AK (2016). Where hearing starts: the development of the mammalian cochlea. J. Anat..

[49] Birch HL (2018). Extracellular matrix and ageing. Subcell. Biochem..

[50] Dong Y, Guo CR, Chen D, Chen SM, Peng Y, Song H, Shi JR (2018). Association between age-related hearing loss and cognitive decline in C57BL/6J mice. Mol. Med. Rep..

[51] Ohlemiller KK (2006). Contributions of mouse models to understanding of age- and noise-related hearing loss. Brain Res..

[52] Noben-Trauth K, Zheng QY, Johnson KR (2003). Association of cadherin 23 with polygenic inheritance and genetic modification of sensorineural hearing loss. Nat. Genet..

[53] Keithley EM (2020). Pathology and mechanisms of cochlear aging. J. Neurosci. Res..

[54] Keithley EM, Canto C, Zheng QY, Fischel-Ghodsian N, Johnson KR (2004). Age-related hearing loss and the ahl locus in mice. Hear Res..

[55] Johnson KR, Erway LC, Cook SA, Willott JF, Zheng QY (1997). A major gene affecting age-related hearing loss in C57BL/6J mice. Hear Res..

[56] Keikhosravi A, Liu Y, Drifka C, Woo KM, Verma A, Oldenbourg R, Eliceiri KW (2017). Quantification of collagen organization in histopathology samples using liquid crystal based polarization microscopy. Biomed. Opt. Express.

[57] Lim DJ, Kim HN (1983). The canaliculae perforantes of Schuknecht. Adv. Oto-Rhino-Laryngol..

[58] Ehret G (1978). Stiffness gradient along the basilar membrane as a basis for spatial frequency analysis within the cochlea”. J. Acoust. Soc. Am..

[59] Ouellette JN, Drifka CR, Pointer KB, Liu Y, Lieberthal TJ, Kao WJ, Kuo JS, Loeffler AG, Eliceiri KW (2021). Navigating the collagen jungle: the biomedical potential of fiber organization in cancer. Bioengineering.

[60] Walters BJ, Zuo J (2013). Postnatal development, maturation and aging in the mouse cochlea and their effects on hair cell regeneration. Hear Res..

[61] Bhatt KA, Liberman MC, Nadol JB (2001). Morphometric analysis of age-related changes in the human basilar membrane. Ann. Otol. Rhinol. Laryngol..

[62] Robin NH, Moran RT, Ala-Kokko L, Adam MP (2000). Stickler syndrome. GeneReviews®.

[63] Nozu K, Nakanishi K, Abe Y, Udagawa T, Okada S, Okamoto T, Kaito H, Kanemoto K, Kobayashi A, Tanaka E, Tanaka K, Hama T, Fujimaru R, Miwa S, Yamamura T, Yamamura N, Horinouchi T, Minamikawa S, Nagata M, Iijima K (2019). A review of clinical characteristics and genetic backgrounds in Alport syndrome. Clin. Exp. Nephrol..

[64] Theocharis AD, Skandalis SS, Gialeli C, Karamanos NK (2016). Extracellular matrix structure. Adv. Drug Deliv. Rev..

[65] Choong JK, Hampson AJ, Brody KM, Lo J, Bester CW, Gummer AW, Reynolds NP, O'Leary SJ (2020). Nanomechanical mapping reveals localized stiffening of the basilar membrane after cochlear implantation. Hear Res..

[66] Muncie JM, Weaver VM (2018). The physical and biochemical properties of the extracellular matrix regulate cell fate. Curr. Top. Dev. Biol..

[67] Lansky Z, Mutsafi Y, Houben L, Ilani T, Armony G, Wolf SG, Fass D (2019). 3D mapping of native extracellular matrix reveals cellular responses to the microenvironment. J. Struct. Biol..

[68] Yang W, Vethanayagam RR, Dong Y, Cai Q, Hu BH (2015). Activation of the antigen presentation function of mononuclear phagocyte populations associated with the basilar membrane of the cochlea after acoustic overstimulation. Neuroscience.

[69] Frye MD, Yang W, Zhang C, Xiong B, Hu BH (2017). Dynamic activation of basilar membrane macrophages in response to chronic sensory cell degeneration in aging mouse cochleae. Hear Res..

[70] He W, Yu J, Sun Y, Kong W (2020). Macrophages in noise-exposed cochlea: changes, regulation and the potential role. Aging Dis..

[71] Guo B, Guo Q, Wang Z, Shao JB, Liu K, Du ZD, Gong SS (2020). D-Galactose-induced oxidative stress and mitochondrial dysfunction in the cochlear basilar membrane: an in vitro aging model. Biogerontology.

[72] Lukashkina VA, Levic S, Lukashkin AN, Strenzke N, Russell IJ (2017). A connexin30 mutation rescues hearing and reveals roles for gap junctions in cochlear amplification and micromechanics. Nat. Commun..

[73] Cogliati B, Vinken M, Silva TC, Araújo C, Aloia T, Chaible LM, Mori C, Dagli M (2015). Connexin 43 deficiency accelerates skin wound healing and extracellular matrix remodeling in mice. J. Dermatol. Sci..

[74] Bonnans C, Chou J, Werb Z (2014). Remodelling the extracellular matrix in development and disease. Nat. Rev. Mol. Cell Biol..

[75] Hynes RO (2009). The extracellular matrix: not just pretty fibrils. Science.

[76] Humphrey JD, Dufresne ER, Schwartz MA (2014). Mechanotransduction and extracellular matrix homeostasis. Nat. Rev. Mol. Cell Biol..

[77] Corns LF, Johnson SL, Roberts T, Ranatunga KM, Hendry A, Ceriani F, Safieddine S, Steel KP, Forge A, Petit C, Furness DN, Kros CJ, Marcotti W (2018). Mechanotransduction is required for establishing and maintaining mature inner hair cells and regulating efferent innervation. Nat. Commun..

[78] Jeng JY, Ceriani F, Hendry A, Johnson SL, Yen P, Simmons DD, Kros CJ, Marcotti W (2020). Hair cell maturation is differentially regulated along the tonotopic axis of the mammalian cochlea. J. Physiol..

[79] Cohen R, Amir-Zilberstein L, Hersch M, Woland S, Loza O, Taiber S, Matsuzaki F, Bergmann S, Avraham KB, Sprinzak D (2020). Mechanical forces drive ordered patterning of hair cells in the mammalian inner ear. Nat. Commun..

[80] Berekméri E, Fekete Á, Köles L, Zelles T (2019). Postnatal development of the subcellular structures and purinergic signaling of deiters' cells along the tonotopic axis of the cochlea. Cells.

[81] Fettiplace R, Nam JH (2019). Tonotopy in calcium homeostasis and vulnerability of cochlear hair cells. Hear Res..

[82] Jeng JY, Ceriani F, Hendry A, Johnson SL, Yen P, Simmons DD, Kros CJ, Marcotti W (2020). Hair cell maturation is differentially regulated along the tonotopic axis of the mammalian cochlea. J. Physiol..

