# Supplementary material for: Postnatal structural development of mammalian Basilar Membrane provides anatomical basis for the maturation of tonotopic maps and frequency tuning
Source: Sci Rep. 2021 Apr 7;11:7581. doi: 10.1038/s41598-021-87150-w (PMC8027603; doi:10.1038/s41598-021-87150-w)
Supplement: Supplementary file 1 — Supplementary Information 1. [file 41598_2021_87150_MOESM1_ESM.docx]

**Title:**

**Postnatal structural development of mammalian Basilar Membrane provides anatomical basis for the maturation of tonotopic maps and frequency tuning**

**Abbreviated title**: Development of Basilar Membrane

**Authors** :

Tomomi Tani^1,2^ ,Maki Koike-Tani^1,3^, Mai Thi Tran^1,4^ , Michael Shribak^1^, Snezana Levic ^1,5,6*^

**Affiliations:**

^1^Eugene Bell Center, Marine Biological Laboratory of University of Chicago, Woods Hole, MA, USA

^2^Biomedical Research Institute, National Institute of Advanced Industrial Science and Technology, Ikeda, Osaka, Japan

^3^Integrated Frontier Research for Medical Science Division, Institute for Open and Transdisciplinary Research Initiatives (OTRI), Osaka University, Suita, Osaka, Japan

^4^College of Engineering and Computer Science, VinUniversity, Gia Lam District, Hanoi, Vietnam.

^5^Sensory Neuroscience Research Group, School of Pharmacy and Biomolecular Sciences, University of Brighton, Huxley Building, Brighton, BN2 4GJ, UK

^6^Brighton and Sussex Medical School, University of Sussex, Brighton BN1 9PX, UK.

Supplementary Figure 1.

**Figure S1. A differential polychromatic polarization image of the organ of Corti** .

1. This is a single-shot instant image, which was captured by polychromatic polarization microscope (PPM) in white light, and color CCD camera Olympus DP73. The PPM was installed in inverted microscope Olympus IX81 equipped with 10x objective lens. DP73 camera has a sensor 1600 x 1200 pixels with pixel size 4.4µm. Then a distance 30µm in the specimen plane corresponds to 68 pixels (=30µmx10/4.4µm). The PPM was optimized for retardance 15 nm. The image hue is almost linearly proportional to the slow axis orientation, and the color saturation is linearly proportional to retardance. 100-µm scale bar (227 pixels).
2. A bright-field polychromatic polarization image, which was cleaned from surrounded dust particles. The differential polychromatic polarization image and LC-Polscope image reveal the birefringent structures only. The bright-field polychromatic polarization image represents a conventional bright-field with added birefringence in color. Thus, one could also see non-birefringent morphological structures.

Supplmentary Figure 2

**Figure S2.** **Sample images collected 15 μm apart showing that LC-pol scope can detect the structural differences at different optical sections.** Images of the basal end of the adult mouse basilar membrane at two different optical sections (15 μm apart) showing that the filament distribution shows spatial difference, collected using LC-PolScope. The brightness represents retardance values, with white representing 12nm retardance and black zero.

**Supplementary movie:**  Movie showing the model of developmental changes in the basilar membrane structure, as revealed from the imaging data.

Please see attached file
